# Tissue Tropism and Target Cells of NSs-Deleted Rift Valley Fever Virus in Live Immunodeficient Mice

**DOI:** 10.1371/journal.pntd.0001421

**Published:** 2011-12-06

**Authors:** Céline Gommet, Agnès Billecocq, Grégory Jouvion, Milena Hasan, Tânia Zaverucha do Valle, Laurent Guillemot, Charlène Blanchet, Nico van Rooijen, Xavier Montagutelli, Michèle Bouloy, Jean-Jacques Panthier

**Affiliations:** 1 Central Animal Facilities, Institut Pasteur, Paris, France; 2 Molecular Genetics of Bunyaviruses, Institut Pasteur, Paris, France; 3 Human Histopathology and Animal Models, Institut Pasteur, Paris, France; 4 Center for Human Immunology, Institut Pasteur, Paris, France; 5 Mouse Functional Genetics, Institut Pasteur, Paris, France; 6 Centre National de la Recherche Scientifique, Unité de Recherche Associée 2578, Paris, France; 7 Cytokines and Inflammation, Institut Pasteur, Paris, France; 8 Department of Molecular Cell Biology, Vrije Universiteit Medical Center, Amsterdam, The Netherlands; Centers for Disease Control and Prevention, United States of America

## Abstract

**Background:**

Rift Valley fever virus (RVFV) causes disease in livestock and humans. It can be transmitted by mosquitoes, inhalation or physical contact with the body fluids of infected animals. Severe clinical cases are characterized by acute hepatitis with hemorrhage, meningoencephalitis and/or retinitis. The dynamics of RVFV infection and the cell types infected *in vivo* are poorly understood.

**Methodology/Principal Findings:**

RVFV strains expressing humanized *Renilla* luciferase (hRLuc) or green fluorescent protein (GFP) were generated and inoculated to susceptible *Ifnar1*-deficient mice. We investigated the tissue tropism in these mice and the nature of the target cells *in vivo* using whole-organ imaging and flow cytometry. After intraperitoneal inoculation, hRLuc signal was observed primarily in the thymus, spleen and liver. Macrophages infiltrating various tissues, in particular the adipose tissue surrounding the pancreas also expressed the virus. The liver rapidly turned into the major luminescent organ and the mice succumbed to severe hepatitis. The brain remained weakly luminescent throughout infection. FACS analysis in RVFV-GFP-infected mice showed that the macrophages, dendritic cells and granulocytes were main target cells for RVFV. The crucial role of cells of the monocyte/macrophage/dendritic lineage during RVFV infection was confirmed by the slower viral dissemination, decrease in RVFV titers in blood, and prolonged survival of macrophage- and dendritic cell-depleted mice following treatment with clodronate liposomes. Upon dermal and nasal inoculations, the viral dissemination was primarily observed in the lymph node draining the injected ear and in the lungs respectively, with a significant increase in survival time.

**Conclusions/Significance:**

These findings reveal the high levels of phagocytic cells harboring RVFV during viral infection in *Ifnar1*-deficient mice. They demonstrate that bioluminescent and fluorescent viruses can shed new light into the pathogenesis of RVFV infection.

## Author Summary

Rift Valley fever, caused by a member of the *Bunyaviridae* family, has spread during recent years to most sub-Saharan African countries, in Egypt and in the Arabian peninsula. The virus can be transmitted by insect vectors or by direct contacts with infectious tissues. The analysis of virus replication and dissemination in laboratory animals has been hampered by the need to euthanize sufficient numbers of animals and to assay appropriate organs at various time points after infection to evaluate the viral replication. By following the bioluminescence and fluorescence of Rift Valley fever viruses expressing light reporters, we were able to track the real-time dissemination of the viruses in live immunodeficient mice. We showed that the first infected organs were the thymus, spleen and liver, but the liver rapidly became the main location of viral replication. Phagocytes also appeared as important targets, and their systemic depletion by use of clodronate liposomes decreased the number of viruses in the blood, delayed the viral dissemination and prolonged the survival of the infected mice.

## Introduction

Rift Valley fever virus (RVFV) is an arthropod-borne member of the *Bunyaviridae* family, genus *Phlebovirus* that causes recurrent outbreaks affecting humans and animals. The virus can be transmitted by *Aedes* and *Culex* mosquitoes [1], although it can also be transmitted by inhalation or physical contact with the body fluids from infected animals [2], [3]. Identified in the 1930s in Kenya, RVFV has spread during recent years to most sub-Saharan African countries, in Egypt and in the Arabian Peninsula, and in the Indian Ocean islands of Grande Comore and Mayotte [4], [5], [6]. In humans, RVFV infections are generally either asymptomatic or characterized by a feverish syndrome without any severe sequelae. However, a small percentage of patients exhibit complications, characterized by acute hepatitis with hemorrhage, meningoencephalitis and/or retinitis [7], [8], [9], [10]. A relationship has been demonstrated between high viral load in blood and death of the patient [11], [12]. RVFV infects domestic ruminants, including sheep, cattle, goats, and camels. It is responsible for massive abortion events in pregnant ruminants and high mortality in lambs and calves. High viremia associated with hepatic necrosis and increase of liver enzymes are hallmarks of severe acute lethal infection in ruminants [13], [14]. Encephalomyelitis has been described in calves [15]. Laboratory rodents such as mice are also highly susceptible to RVFV infection. In outbred Swiss mice, the survival time was inversely proportional to the logarithm of the viral dose inoculated via the intravenous route [16]. Depending on their genotype, males from various inbred strains of mice inoculated by the peritoneal route with 10^2^ PFU of the virulent Egyptian ZH548 strain die between 4 to 10 days after inoculation, illustrating natural variation in susceptibility of the host to RVF [17]. The main damages of mouse infection with RVFV can be observed early in the liver, with extensive apoptosis of hepatocytes, accompanied in the blood by a peak in liver enzymes, along with increased bilirubin levels [18], [19], [20]. It has been recently shown that mice that survive hepatitis develop later infection of the brain, and eventually die from meningoencephalitis [21]. Interestingly, a diverse set of cell types from a number of tissues was found to contain RVFV antigens, including mononuclear phagocytes, but also cardiac myofibers, pancreatic islet cells, and adrenal medullary cells [21]. These data showed that RVFV exhibits a large tropism for a variety of tissues and individual cell types. Quantitative real-time PCR have also been used to study the kinetics of RVFV infection in the blood and organs of infected mice [22]. High amounts of RVFV RNA were found in blood, liver and brain samples shortly after infection with the highest viral RNA levels in the liver. We hypothesized that *in vivo* imaging might be an alternative method to assess viral replication using a recombinant RVFV carrying a reporter gene that allows the monitoring of viral expression in live animal.

RVFV has a tripartite negative-sense, single-stranded RNA genome with large (L), medium (M) and small (S) segments. The L segment encodes the viral RNA-dependent RNA polymerase, the M segment the two virion glycoproteins (G_N_ and G_C_) and the NSm nonstructural proteins, and the S segment the N nucleoprotein and the NSs nonstructural protein. Reverse genetic systems have been successfully developed for the recovery of recombinant RVFV (reviewed in [23]). These rescue systems rely on transfection with plasmids expressing the three viral RNAs, and the N nucleoprotein and L RNA-dependent RNA polymerase, which are required for the packaging and replication of the viral RNAs. The RVFV RNA genome segments are expressed under the control of either the cellular DNA-dependent RNA polymerase I promoter [24], [25], [26] or the bacteriophage T7 promoter in cells that constitutively express T7 RNA polymerase [24]. Such rescue systems have been used to produce recombinant RVFV, including various mutants that lack the NSs, NSm genes, or carry specific mutations [26], [27], [28], [29], [30]. Viral strains that express reporter genes were also generated [24], [31], [32]. Importantly, in these recombinant viruses, the reporter gene activity directly reflects the extent of both viral transcription and replication.

In this study, we aimed to detect and quantify viral replication in living animals using two recombinant RVFV strains expressing either humanized version of the luciferase gene of *Renilla reniformis* (hRLuc) or enhanced green fluorescent protein (GFP) gene of *Aequora victoria*. Both RVFV viruses lack a functional NSs gene, which is a main factor of virulence in mice [33], and are therefore avirulent in immunocompetent mice. However, in mice that are nonresponsive to type I IFN, the virus expressing either hRLuc or GFP caused lethality within 3 days, in agreement with previous data for the NSs-deficient Clone 13 [24]. In these mice, virus infection could be tracked by luciferase imaging in live animals and by the detection of GFP-positive cells from infected animals, by use of flow cytometry. We observed qualitative and quantitative differences in the *in vivo* tropism of RVFV in mice and show previously unsuspected sites of virus replication and modes of virus spread.

## Methods

### Mice, cells and virus

Animals were housed in the Institut Pasteur animal facilities accredited by the French Ministry of Agriculture to perform experiments on live mice, in appliance of the French and European regulations on care and protection of the Laboratory Animals (accreditation number B 75 15-01 and B 75 15-07). The veterinary staff of the Institut Pasteur animal facility approved protocols. Protocols were performed in compliance with the NIH Animal Welfare Insurance #A5476-01 issued on 02/07/2007. Inbred 129S2/SvPas mice with knockout at the interferon α and β receptor 1 locus (*Ifnar1^−/−^*) and control mice (*Ifnar1* ^+/+^) were bred at the Institut Pasteur [34]. Vero E6 cells were grown in DMEM supplemented with 10% FCS. BHK21/T7 cells [35] were grown in MEM supplemented with 5% FCS and tryptose phosphate broth powder (Sigma-Aldrich, Gillingham, UK). The cell culture media were supplemented with 10 IU/ml of penicillin and 10 µg/ml of streptomycin. Stocks of the virulent RVFV Egyptian ZH548 strain were produced under biosafety level 3 (BSL3) conditions.

### Production of recombinant RVFV strains

Plasmids pPol I-LZH, pPol I-MZH, and pPol I-SZH carrying the L, M and S segments of ZH548, respectively, were cloned in the plasmid pRF108, [24], [36]. The plasmid pPol I-SZHΔNSs, derived from pPol I-SZH, carries two *Bbs*I cloning sites in place of NSs [24]. The humanized *Renilla reniformis* luciferase sequence from phRL-SV40 (Promega, Charbonnières-les-Bains, France) was inserted in pPol I-SZHΔNSs to give pPol I-SZHΔNSs-hRLuc. The structure of pPol I-SZHΔNSs-hRLuc plasmid was confirmed by sequence analysis. Recombinant rZHΔNSs-GFP [24] and rZHΔNSs-hRLuc RVFV stocks were produced under BSL3 conditions. Approximately 5×10^5^ BHK21/T7 cells were seeded in triplicate in 35 mm culture dishes. The following day, they were combined with FuGENE®6 transfection reagent (Roche Applied Science, Indianapolis, IN) and 0.5 µg each of pTM1-L and pTM1-N [25], and 1 µg each of pPol I-LZH, pPol I-MZH, and either pPol I-SZHΔNSs-GFP or pPol I-SZHΔNSs-hRLuc in OptiMEM (Gibco, invitrogen, Carslbad, CA). One day later, the medium was renewed. Five days later, the supernatant containing the rescued virus was collected and stored at −80°C. To produce viral stocks of rZHΔNSs-GFP and rZHΔNSs-hRLuc, Vero E6 cells were infected with the rescued virus at a MOI of 0.001 and 0.01, respectively. At 72 h post-infection, the supernatant was collected and the viral suspension was titered.

### Virus titration by plaque assay

Vero E6 cells, infected with serial dilutions of viral suspension, were incubated under an overlay of DMEM supplemented with 2% FCS, antibiotics and 1% agarose. Four days later, the plates were stained with 0.2% crystal violet in 10% formaldehyde, 20% ethanol and the lytic plaques were counted.

### hRLuc expression analysis *in vitro*

To test the luciferase expression within cells after infection with rZHΔNSs-hRLuc, Vero E6 cells were infected using a MOI of 0.3 and 3. Next, every 2 h, for 12 h, luciferase activity was measured in triplicates by Renilla Luciferase Assay System (Promega, Madison, WI). To check the stability of luciferase expression through passages, Vero E6 cells were infected using an MOI of 3 and the luciferase activity was measured in triplicates at 8 h post-infection.

### GFP and viral expression analysis *in vitro*

To test the GFP expression, Vero E6 cells were infected with rZHΔNSs-GFP using a MOI of 1. At 15 h post-infection, the cells were fixed for 30 min at room temperature with 4% paraformaldehyde in PBS, permeabilized for 10 min with 0.5% Triton X100 in PBS and incubated for 30 min at room temperature in 5% bovine serum albumin (BSA) in PBS. The cells were next incubated for 30 min at 37°C with a mouse anti-N antibody diluted in 5% BSA in PBS (dilution 1∶800), washed with 5% BSA in PBS and incubated for 25 min at 37°C with the secondary antibody Alexa Fluor 555 goat anti-mouse (Invitrogen, Paisley, UK) diluted in 5% BSA in PBS (dilution 1∶1200). Finally, the cells were washed with 5% BSA in PBS and then in water. The slides were mounted with Fluoromont-G (SouthernBiotech, Birmingham, AL). The cells were observed under an Axioplan 2 Imaging microscope (Zeiss, Le Pecq, France) using excitation and emission filter allowing simultaneous detection of GFP and Alexa Fluor 555.

### Infection of mice and monitoring

One week prior to infection, five to six week-old mice were transferred in BSL3 isolators to allow acclimatization. After this period, they were inoculated intraperitoneally (i.p.), intradermally (i.d.) or intranasally with 10^4^ PFU ZH548, rZHΔNSs-hRLuc or rZHΔNSs-GFP RVFV in DMEM supplemented with 2% FCS, and antibiotics. For i.p. infection, mice were inoculated with 100 µL of viral suspension. For i.d. and intranasal infections, mice were first anesthetized with ketamine (150 mg/kg) and xylazine (10 mg/kg) administered i.p., then inoculated with either 15 to 30 µL or 15 µL of viral suspension into the ear (i.d.) or intranasally, respectively. Mortality was recorded at least twice a day from day 1 to 4 post-infection and once a day after day 5 post-infection until the end of the observation period. Animals were observed for a maximum of 14 days.

### *In vivo* bioluminescence imaging

To improve bioluminescence imaging, hairs were removed [37]. We observed the mice to detect clinical signs due to the infection prior to imaging. Mice that exhibited no severe clinical signs were anesthetized and injected i.p. with 100 µL h-coelenterazine (1 mg/ml). The h-coelenterazine stock solution provided by Nanolight Technology (Pinetop, AZ) was solubilized in ethanol-propylene glycol solution (1∶1) at 10 mg/ml. This solution was diluted in PBS (1∶9) just before imaging. The h-coelenterazine-treated mice were immediately placed in a hermetically sealed light-tight-transparent chamber (TEM Sega, Lormont, France) equipped with two HEPA filters. One HEPA filter was connected to an air pump, thus allowing air renewal during the imaging. The bio containment chamber allowed simultaneous imaging of 6 mice. The mice were imaged 15 and 20 min after h-coelenterazine injection for the whole body and the thorax, respectively [38]. Imaging was performed with a Xenogen's IVIS 100 system, including a cooled charge-coupled device (CCD) camera [39], [40]. Integration periods ranged from 0.5 to 120 s depending on the amounts of light emitted at various infection sites. Images were obtained using Living Image® 3.1 software (Xenogen, Alameda, CA). Specific regions of interest (ROI) on the images were defined without overlay, using the anatomic location of the different organs and their visual observation through the skin when possible. For each imaging session, a mock-infected mouse was used as a negative control. A signal was considered significant if its intensity in infected mice was at least twofold higher than the background luminescence in the mock-infected mouse. After the last imaging time point, mice were euthanized.

### *Ex vivo* bioluminescence imaging

For *ex vivo* imaging, selected mice were euthanized at different times after infection for harvest of the following organs: liver, spleen, thymus, lung, kidney, stomach, small and large intestine, heart, ovary and uterus, testis, epididymis, seminal vesicles and preputial glands. The organs were placed in 6 (intestine) or 2 ml (all other organs) of PBS. Before imaging, 1 µL/ml h-coelenterazine 5 mM in ethanol and propylene glycol (1∶1) was added [41]. Imaging was performed in a hermetically sealed chamber to avoid light. Images were acquired 10 min after the addition of h-coelenterazine. Integration period ranged from 0.5 to 60 s depending on the amounts of light emitted from various organs.

### RNA extraction and quantitative RT-PCR

RNA was extracted using Trizol LS reagent (Invitrogen, Carslbad, CA) and suspended in RNase free water. RNA was quantified using Nanodrop 3300 (Thermo Scientific, Courtaboeuf, France). The M segment of RVFV was amplified with primers 5′-CATGGATTGGTTGTCCGATCA-3′ and 5′-TGAGTGTAATCTCGGTGGAAGGA-3′. Quantitative RT-PCR assays were performed using StepOne Plus Real-Time PCR System (Applied Biosystem, Courtaboeuf, France) in 96-well plates. Reverse transcription using MultiScribe Reverse Transcriptase (Applied Biosystem) at 48°C for 30 min was performed followed by a standard amplification program. The size of the amplification product was 108 pb. A standard curve was generated using duplicates of 10-fold serial dilutions of RNA of the M segment ranging from 10^9^ to 10^2^ copies. Quantification of viral RNA was done by comparison of the threshold cycle (Ct) values of the samples to the standards.

### Histopathological analysis

Histopathological and immunohistochemical analysis of wild-type mice infected with 10^4^ PFU ZH548 RVFV was performed 3 to 5 days after i.p. inoculation. rZHΔNSs-hRLuc-infected *Ifnar1^−/−^*mice were euthanized at 8, 16 and 34 h after i.p. inoculation. For each time point, a complete post-mortem examination was carried out. The lung, brain, kidneys, spleen, liver, pancreas, thymus, testis, uterus and ovaries were removed and immediately fixed for one week in 10% neutral buffered formalin. Samples from each organ were embedded in paraffin and five-micrometer sections were then cut and stained with hematoxylin and eosin (HE). The histological characterization of lesions was completed by an immunohistochemical detection of the RVFV using mouse antibodies against the RVFV (dilution 1∶100) visualized with the Histofine Simple Stain MAX-PO kit (Histofine Biosciences inc, Cambridge, UK).

### Flow cytometric analysis

Eleven *Ifnar1*-deficient 129S2/SvPas mice were either infected i.p. with 10^4^ PFU rZHΔNSs-GFP RVFV (N = 6) or mock-infected (N = 5). Twenty-four hours later, the spleen was harvested. Erythrocytes were lysed using NH_4_Cl (9 g/L) buffer. The rat anti-mouse CD16/CD32, clone 2.4G2 antibody (BD Pharmingen, San José, CA) was used to block non-antigen-specific binding of immunoglobulins to Fc-receptors. Cells were stained using a combination of the following antibodies: (i) PE-conjugated rat anti-mouse NKp46/CD335 (BD Pharmingen). (ii) PerCP-Cy5.5-conjugated hamster anti-mouse CD3 (BD Pharmingen). (iii) APC-conjugated rat anti-CD19 (BD Pharmingen). (iv) Pacific Blue-conjugated rat anti-mouse CD11b/Mac-1 (eBioscience, San Diego, CA). (v) APC-conjugated hamster anti-mouse CD11c/Itgax (BD Pharmingen). (vi) Alexa Fluor 700-conjugated rat anti-mouse MHC Class II (I-A/I-E) (eBioscience). (vii) PE-conjugated rat anti-mouse Ly6G/Gr-1 (BD Pharmingen). (viii) Biotin-conjugated anti-mouse CD115/c-Fms (eBioscience) with Streptavidin-PerCP-Cy5.5 (BD Pharmingen) as second-step reagent. All staining procedures were conducted on ice. Then, the cells were fixed with 4% formaldehyde. Fluorescence was measured using a FACSAria II flow cytometer (BD Biosciences, San Jose, CA), and data analysis was performed using CellQuest (BD Biosciences) and FlowJo (Ashland, OR) softwares. Dead cells were visualized using the Fixable Aqua Dead Cell Stain kit (Invitrogen, Carlsbad, CA). Fluorescence compensation settings for multicolor flow cytometric analysis were optimized based on single-stained polystyrene microparticles (CompBeads, BD Pharmingen).

### Liposome treatment

Clodronate (Cl2MBP; dichloromethylene-biphosphonate)-loaded liposomes (CLL) were used to deplete phagocytic cells [42], [43]. Clodronate was a gift of Roche Diagnostics GmbH, (Mannheim, Germany). It was encapsulated in liposomes as described earlier [42], [43]. Mice were injected i.p. with 300 µL and i.v. with 200 µL CLL. Control mice were treated i.p. and i.v. with PBS-loaded liposomes. Twenty-four hours later, single-cell suspensions were prepared from blood and spleen. FcR blocking reagent mouse (Miltenyi Biotec, Bergisch Gladbach, Germany) was used to block non-antigen-specific binding of immunoglobulins to Fc-receptors. Cells were stained using combination of the following antibodies: FITC-conjugated rat anti-mouse CD11b (BD Pharmingen), PE-conjugated rat anti-mouse CD115 (eBioscience.com), APC-conjugated rat anti-mouse F4/80 (eBioscience.com), PE-conjugated hamster anti-mouse CD11c (BD Pharmingen) and Pacific Blue-conjugated rat anti-mouse Ly6G/Gr-1 (eBioscience.com). All staining procedures were conducted on ice. Fluorescence data were obtained and analyzed using MACSQuant Analyzer and MACSQuantify software (Miltenyi Biotec). Challenge with 10^4^ PFU rZHΔNS-hRLuc was performed by injection into the ear, 24 h after liposome treatment.

### Statistical analysis

The survival curves were compared using the logrank test. The bioluminescence signals and blood plasma viral loads were analyzed with the nonparametric Mann-Whitney test. All tests were performed using the StatView 5.0 software (SAS Institute Inc, Cary, NC).

## Results

### Production and characterization of recombinant Rift Valley fever viruses

**Figure 1:**
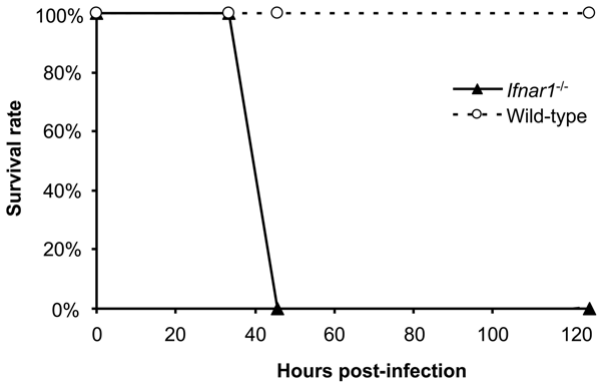
Survival curve of mice challenged with rZHΔNSs-hRLuc RVFV. *Ifnar1*-deficient and wild-type mice were infected i.p. with 10^4^ PFU rZHΔNSs-hRLuc virus. Mice were monitored twice daily for 4 days and, later, once daily for up to 13 days post-infection. *Ifnar1*-deficient mice succumbed to disease between 40 and 50 hours post-infection (N = 10). Wild-type 129S2/SvPas mice challenged with rZHΔNSs-hRLuc did not succumbed to infection (N = 5).

The generation of a recombinant RVFV expressing a green fluorescent protein (GFP), rZHΔNSs-GFP, has previously been described [24]. Our previous attempts to generate a recombinant RVFV expressing a humanized firefly luciferase (hFLuc) have been confounded by genetic instability and the rapid emergence of mutants with deletions [24]. Therefore, we generated rZHΔNSs-hRLuc RVFV that carries a humanized *Renilla* luciferase (hRLuc) gene using Pol I based plasmids as previously described [24]. The rescued rZHΔNSs-hRLuc was amplified in Vero E6 cells and stocks produced. The titer reached 8×10^7^ PFU/ml. The plaques formed by rZHΔNSs-hRLuc were fuzzy with a faint staining inside, as those obtained with the rZHΔNSs virus that carries a deletion of the NSs gene [24]. To test the luciferase expression within the infected cells, Vero E6 cells were infected with rZHΔNSs-hRLuc at a MOI of either 0.3 or 3, and lysed every 2 hours for 12 h. Luciferase activity was measured using coelenterazine, a specific substrate of *Renilla* luciferase, and found to be expressed at significant levels from 2 h post-infection onwards, while uninfected Vero E6 cells showed no luciferase activity. The luciferase activity increased with time and was dependent on the MOI (data not shown). This is consistent with previous reports [31], [32]. To check the stability of the recombinant virus *in vitro*, rZHΔNSs-hRLuc was passaged on Vero E6 cells and, at each passage, we measured the viral titer in the supernatant at 72 h post-infection and the luciferase activity within the infected cells at 8 h post-infection with a MOI of 3. Both the viral titer and the luciferase activity remained stable over at least 8 passages, varying from 10^7^ to 10^8^ PFU/ml and from 10^7^ to 10^8^ raw light units (RLU)/s per 3×10^5^ cells, respectively. To test the virulence of the recombinant virus, wild-type 129S2/SvPas mice (N = 5) and 129S2/SvPas mice deficient for IFN-α/β receptor subunit 1 (*Ifnar1^−/−^*) (N = 10) were infected i.p. with 10^4^ PFU of rZHΔNSs-hRLuc. All wild-type mice survived the infection for 13 days with no signs of disease, as seen following infection with rZHΔNSs in which the NSs gene is totally deleted [24] or Clone 13, a natural isolate that lacks 69% of the NSs open reading frame [33]. In contrast, all rZHΔNSs-hRLuc infected *Ifnar1^−/−^* mice died within 45 h (Figure 1) from severe hepatitis with no signs of neurological disorder. Infections of *Ifnar1^−/−^* mice with rZHΔNSs or with Clone 13 gave similar results ([24] and data not shown).

To evaluate the stability of the recombinant viruses in live animals, total RNAs were extracted from the liver of rZHΔNSs-hRLuc- or rZHΔNSs-GFP-infected *Ifnar1^−/−^* mice at 34 h post-infection and RT-PCR assays were performed using primer pairs flanking the hRLuc or GFP reporter gene. The amplification product sizes were those expected from the structure of pPolI-SZHΔNSs-hRLuc and pPolI-SZHΔNSs-GFP plasmids (data not shown). No amplification products with smaller sizes were observed, suggesting that the recombinant viruses maintained their own genomic stability not only in cultured cells, but also during *in vivo* infection. Furthermore, to examine the reporter expression stability after *in vivo* infection, the recombinant rZHΔNSs-GFP was harvested from the liver of an infected *Ifnar1^−/−^* mouse at 34 h post-infection and then used to infect Vero E6 cells at a MOI of 1. The percentage of cells positive for the N viral protein that were also GFP-positive was measured. The percentage of N-positive, GFP-positive cells was almost identical to that of the initial viral stock (84%±1.90% vs. 85%±7.34%). Altogether, these results suggest that the recombinant viruses were stable for the time of infection in live mice.

### Bioluminescence imaging for Rift Valley fever virus infection

**Figure 2:**
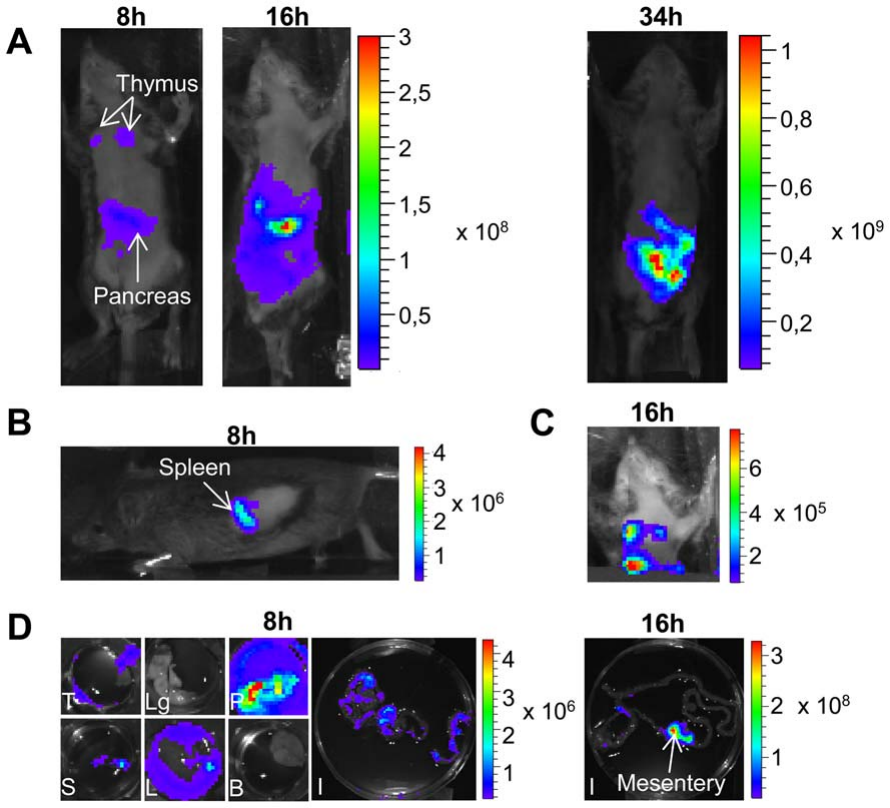
Replication sites of rZHΔNSs-RLuc RVFV following inoculation via the intraperitoneal route. (A) Imaging of an *Ifnar1*-deficient mouse after infection i.p. with 10^4^ PFU rZHΔNSs-hRluc. Imaging was performed at the indicated times post-infection. This image is representative of thirteen mice. (B) Imaging of the left profile of a mouse at 8 h post-infection showing a bioluminescent signal in the spleen. This image is representative of ten mice. (C) Imaging of the thorax at 16 h post-infection of the same mouse as in (A). Occulting the abdominal cavity revealed bioluminescent signal in the thymus. (D) *Ex vivo* imaging after dissection at 8 and 16 h post-infection (T, thymus; Lg, lungs; P, pancreas; S, spleen; L, liver; B, brain; I, intestines). Photographs were overlaid with false-color representations of bioluminescence intensity, measured in photons/s/cm^2^/sr and indicated on the scales.

**Figure 3:**
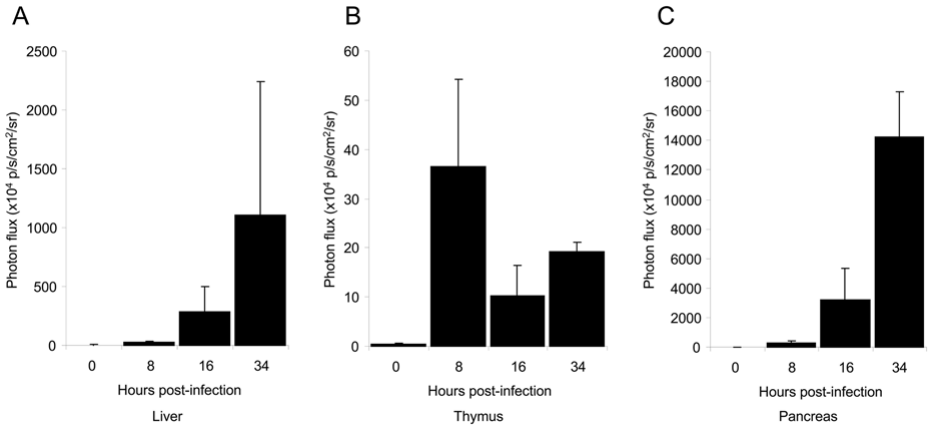
Luciferase levels of the liver, thymus and pancreas during the time course of infection. Five *Ifnar1*-deficient mice were infected i.p. with 10^4^ PFU rZHΔNSs-hRLuc. Imaging of the liver (A), thymus (B) and pancreas (C) was performed in living mice at 8, 16 and 34 h post-infection by defining regions-of-interests (ROIs). Results are given as photon/s/cm^2^/steradian and plotted as the mean and standard error of the mean of photon counts over time.

To visualize the spread of the virus, *Ifnar1^−/−^* mice were infected i.p. with 10^4^ PFU of rZHΔNSs-hRLuc. At 8, 16 and 34 h post-infection, h-coelenterazine was injected i.p. This route of h-coelenterazine administration was preferred to tail-vein injection due to slower kinetics of light production, as previously reported [39]. Mice were observed with real-time *in vivo* imaging 15 and 20 min after the injection of h-coelenterazine for the whole body and the thorax, respectively. At 8 h post-infection, luminescence was readily detected. Short integration periods (15 s) were sufficient to acquire a significant signal. We observed strong signals between the forelegs in the thoracic cavity, and below the xiphoid cartilage in the abdominal cavity, respectively (Figure 2A). Imaging of the left profile showed an additional signal in the spleen (Figure 2B). In some experiments, animals were euthanized for *ex vivo* imaging, the organs of the thorax and abdomen were harvested, and the individual organs were imaged (Figure 2D). In the thorax, the greatest signal originated from the thymus, whereas the signal from the lungs was only slightly above background. In the abdomen, the pancreas was the most luminescent organ. The spleen and the liver also emitted significant luminescence. On average, a ten-fold higher luminescence signal was observed in the harvested pancreas compared to the liver. This suggests that the liver, the critical target organ of the disease, was not among the main replication sites for RVFV at this early stage of infection. At 16 h post-infection, the whole body luminescence was higher than at 8 h post-infection (Figure 2A). The signal spread out the abdominal cavity. The high intensity of luminescence in the abdominal cavity precluded detection in other locations unless integration was limited to the thorax (Figure 2C). Dissection and *ex vivo* imaging showed a gradual increase of luminescence in the pancreas and in the liver. Additional sources of luminescence were the intestine mesentery, kidneys, ovaries and uterus in females, the seminal vesicles, preputial glands, epididymis and testis in males (Figure 2D, and data not shown). The intensity of these signals was quite similar to that measured in the spleen and in the liver (data not shown). At 34 h post-infection, the intensity of the signal led to saturation of the camera using a 0.5 s integration period, thus preventing identification of individual organs (Figure 2A). *Ex vivo* imaging revealed that the highest signal was in the liver. Other organs with intense luminescence were the spleen, intestine mesentery and pancreas (data not shown). Quantification of the luciferase expression in living mice during the time course of infection showed that the luminescence signal originated from the thymus remained constant from 8 h post-infection onwards, whereas luminescence profiles were increased in the liver and pancreas, suggesting a progressive increase of viral replication in these organs (Figure 3).

**Figure 4:**
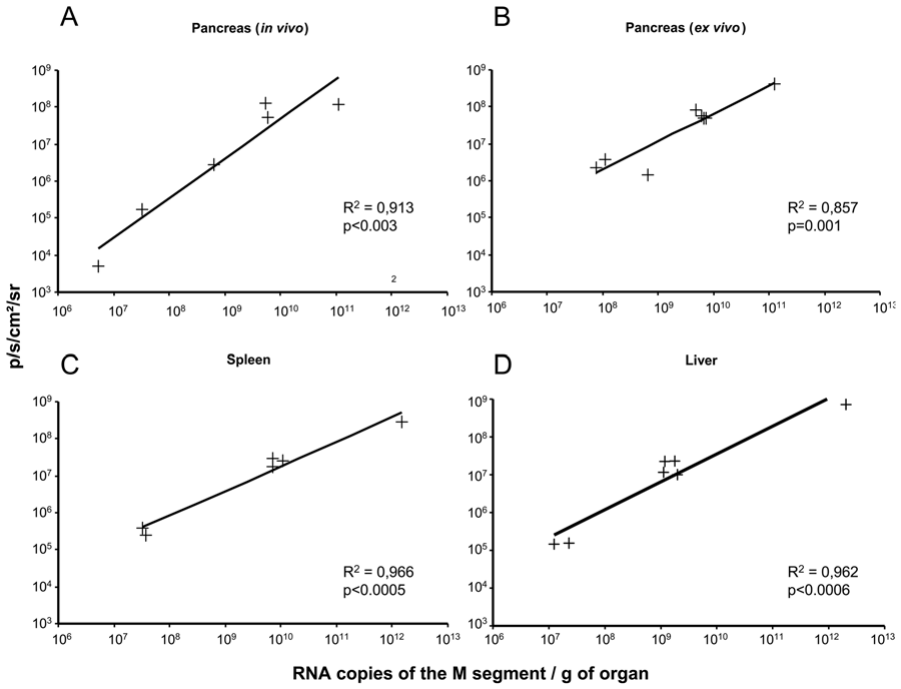
Emitted photons and viral RNA copy number in the pancreas, spleen and liver. Mice were infected i.p with 10^4^ PFU rZHΔNSs-hRLuc. Bioluminescence imaging was performed at 8, 16 and 34 h post-infection. (A) Bioluminescence imaging of the pancreas was performed in anesthetized mice. Subsequently, mice were euthanized and pancreas were harvested and further processed for determination of the viral RNA copy number. (B–D) Mice were euthanized to harvest the pancreas, spleen and liver. The light emission was measured in the harvested pancreas (B), spleen (C) and liver (D) by imaging. Each cross represents an individual organ. Results are given as the number of RNA copies per gram of organ (x axis) with the y axis showing the flux expressed in p/s/cm^2^/sr. R^2^ and *P* indicate the correlation coefficient and the significance level, respectively.

To determine whether there was a correlation between the luminescence detected by the camera and the amount of virus genomes in the tissues, rZHΔNSs-hRLuc-infected *Ifnar1^−/−^* living mice were subjected to imaging. Next, the animals were euthanized and the organs harvested and imaged. Total RNAs were extracted from the organs and RVFV RNA copy numbers were measured by quantitative real time RT-PCR. We observed a highly significant correlation between the luminescence emitted by the pancreas in living mice and RVFV RNA copy number (Figure 4A). Similarly, luminescence intensity significantly correlated with the RVFV RNA copy number in the harvested pancreas, spleen and liver (Figure 4B–D).

The ability of h–coelenterazine to cross the blood-brain barrier is unknown. To determine whether rZHΔNSs-hRLuc can infect the brain, we dissected and soaked the brain in an h-coelenterazine solution and imaged (Figure 2D). At 8 h post-infection, the luminescence intensity was ten-fold higher in the brain from infected mice compared to control (10^4^ photons/second/cm^2^/steradian [p/sec/cm^2^/sr] vs. 10^3^ p/sec/cm^2^/sr), showing that the RVFV replicated in the brain at an early stage. Light emission increased through 16 h and 34 h post-infection to reach 7×10^5^ and 7×10^6^ p/sec/cm^2^/sr, respectively. Importantly, the intensity of luminescence in the brain was ten- to hundred-fold lower compared to the intensities in the thymus, pancreas, spleen, liver, and intestine mesentery, suggesting that the viral load was lower in the brain than in the thoracic and abdominal organs.

**Figure 5:**
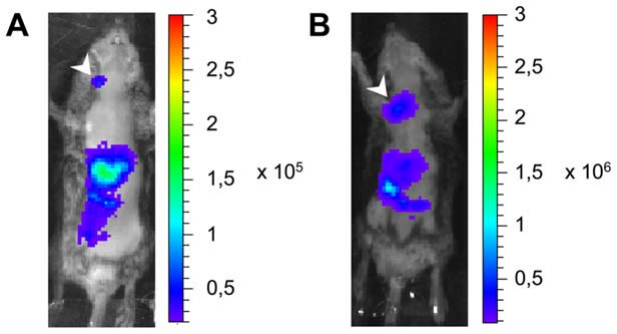
*In vivo* replication sites of rZHΔNSs-RLuc RVFV following inoculation via intradermal or intranasal route. (A) Representative image of luminescence in the lymph node (white arrowhead) draining the right ear of *Ifnar1*-deficient mouse at 24 h after intradermal inoculation of 10^4^ PFU rZHΔNSs-RLuc. (B) Luminescence signal in the lungs (white arrowhead) at 48 h after intranasal inoculation of 10^4^ PFU rZHΔNSs-RLuc. Images (A) and (B) are representative of five mice. Photographs were overlaid with false colour representation of bioluminescence intensity, measured in photon/s/cm^2^/sr and indicated on the scales.

RVFV can be transmitted through injection of infectious saliva from mosquito into the dermis or direct inhalation from body fluids, such as blood of infected animals [2], [3]. To approximate these two natural routes of infection and to monitor their effects, we compared the light production after intraperitoneal, intradermal or intranasal inoculation of 10^4^ PFU rZHΔNSs-hRLuc into *Ifnar1^−/−^* mice. Following intradermal inoculation of the ear pinna (N = 5), luminescence was first visible in the neck on the side of the injected ear, and in the abdominal cavity at 24 h post-infection (Figure 5A). Histologic analysis established the source of the light in the neck; the neck signal came from the lymph nodes draining the injected ear (data not shown). At 40 h post-infection, organs in the abdominal cavity, including the pancreas and the liver were highly luminescent. All mice succumbed to infection by 69 h post-infection, a survival time significantly longer than after i.p. inoculation (*P*<0.025). Luminescent virus inoculated intranasally was already detected 24 h post-infection in the abdomen. This mode of inoculation caused an interstitial pneumonia that initiated as a distinctive luminescence signal in the lungs from 48 h post-infection onwards (Figure 5B). Mice infected intranasally (N = 5) survived significantly longer than after i.p. inoculation (*P*<0.0047); all were dead by 69 h post-infection.

### Identification of hematopoietic target cells by immunohistochemistry and flow cytometry

**Figure 6:**
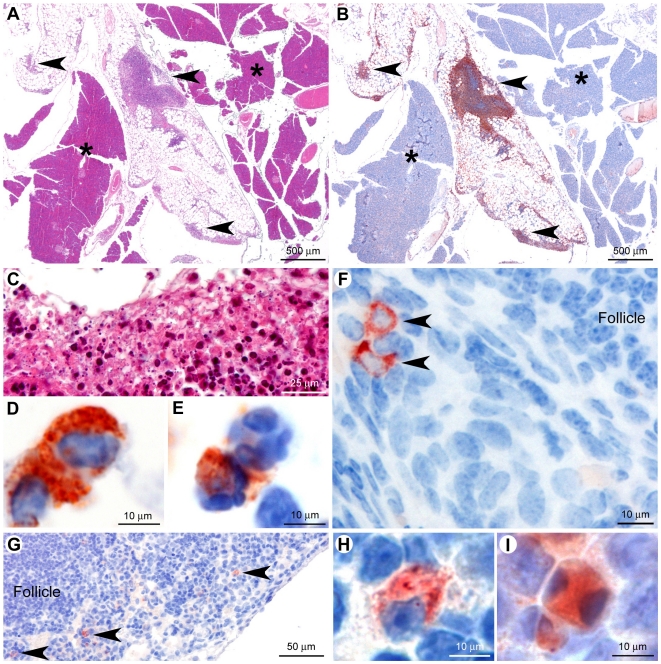
Lesions in the pancreas and expression of viral proteins in the pancreas and ovary. A–F: Histological lesions in *Ifnar1^−/−^* mice infected i.p. with rZHΔNSs-hRLuc RVFV. (A) Multifocal lesion centered on the mesentery, sparing the pancreatic tissue. (B) The expression of viral proteins is seen in the mesentery exclusively (arrowheads). No viral immunolabeling is detected in the endocrine and exocrine components of the pancreas (asterisks). (C) The lesion is characterized by necrosis of mesenteric adipocytes associated with infiltration of macrophages and neutrophils that could be fragmented (suppuration). Note the presence of high number of cell debris in these lesions. (D) Macrophages in the mesentery expressed viral antigens as seen by immunohistochemistry. (E) Neutrophils also expressed viral proteins. Almost all macrophages in the lesions were infected by RVFV. By contrast, neutrophils expressing viral proteins were less numerous. (F) In the ovary, infected macrophages were multifocally detected in the stroma. G–I: Lesions in wild-type 129S2/SvPas mice infected i.p. with ZH548 RVFV strain. (G–H) Multifocal macrophages expressing viral protein were detected in the pancreaticoduodenal lymph node sinus (arrowheads). (I) Some of these macrophages displayed morphological modifications suggesting irreversible injury, including a shrunken cytoplasm and a hyperbasophilic and fragmented nucleus. Hematoxylin and eosin staining (A and C). Immunohistochemistry using antibodies directed against the RVFV (B, and D to I).

To clarify the identity of the RVFV target cells, we carried out histopathological analysis in RVFV-hRLuc-infected *Ifnar1^−/−^* mice at 8, 16 and 34 h after i.p. inoculation. No significant histological lesions were observed at 8 and 16 h post-infection. By contrast, at 34 h post-infection, moderate to marked lesions were detected in the liver, lung, spleen, thymus, ovaries, and in the mesentery surrounding the pancreas (Figure 6, data not shown). No histological lesions were detected in the other organs. In the liver, lung and spleen, the lesions were similar to those previously reported in RVFV-infected wild-type mice [18], [19], [20], [21]. Diffuse apoptosis of lymphoid cells have been previously reported in areas with or without RVFV antigen in the thymus of infected BALB/c mice [21]. Accordingly, we identified the thymus as one of the major targets of RVFV by bioluminescence. However, the pancreas and reproductive organs were also luminescent although none of these organs are known as tissue targets of RVFV. Therefore, to identify cell types that support RVFV replication in these organs, we studied tissue samples by histology and immunohistochemistry with antibodies against the RVFV. In the pancreas, no histological lesions were found in the exocrine or endocrine components (Figure 6A). However, a multifocal inflammatory lesion was observed in the mesentery around pancreatic acini (peritonitis), characterized by necrosis of adipocytes associated with infiltration of macrophages and neutrophils (Figure 6A–C). Viral antigens were present only in the cytoplasm of macrophages (Figure 6D) and, more rarely, in neutrophils (Figure 6E), confirming that the virus did not target the pancreatic exocrine or endocrine cells but macrophages. Similarly, in the ovaries, viral nucleocapsid-positive macrophages were seen in the stroma (Figure 6F). Thus macrophages appeared as important cell targets for the replication of RVFV-hRLuc in *Ifnar1*-deficient mice. To examine whether macrophages are also cell targets for the replication of virulent RVFV in wild-type mice, histopathological and immunohistochemical analysis was performed in wild-type 129S2/SvPas mice (N = 3) infected i.p. with 10^4^ PFU ZH548. Post-mortem analyses were carried out once mice displayed clinical signs, *i.e.* three to five days after the inoculation. Histopathological analysis of the pancreas and its mesentery revealed no peritonitis. However, numerous macrophages containing intracytoplasmic viral antigens were observed in the sinus of the pancreaticoduodenal lymph node (Figure 6, G and H). These macrophages occasionally displayed a hyperbasophilic and condensed nucleus, a morphological change that is characteristic for irreversible cell injury (Figure 6, I). Collectively, these results confirmed that macrophages are important cell targets of the RVFV in the mouse.

**Figure 7:**
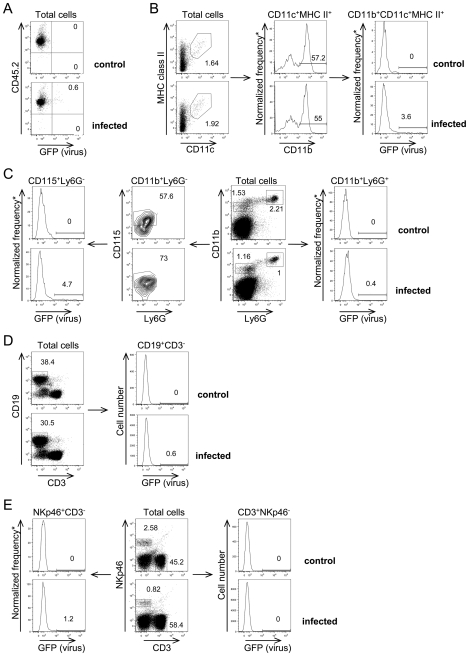
Expression of GFP reporter protein in splenocytes from RVFV-GFP-infected mice. Mice were either infected i.p. with 10^4^ PFU rZHΔNSs-GFP or mock-treated (control) and euthanized 24 h after infection. Splenocytes of rZHΔNSs-GFP-infected mice (lower plots) and control (upper plots) were analyzed by flow cytometry for the expression of GFP. (A) Representative flow cytometry dot plots showing that CD45.2^+^ cells (total hematopoietic cells) of an RVFV-GFP-infected mouse expressed GFP, as evaluated by fluorescence intensity (bottom plot). (B) Histogram showing GFP expression in CD11c^+^ MHCII^+^ CD11b^+^ cells (dendritic cells). (C) Histograms showing GFP expression in Ly6G^−^ CD11b^+^ CD115^+^ cells (macrophages) (left panels) and CD11b^+^ Ly6G^+^ (granulocytes) (right panels). (D) GFP expression was assessed in CD19^+^ CD3^−^ cells (B lymphocytes). (E) GFP expression in NKp46^+^ CD3^−^ cells (NK cells) (left panels) and NKp46^−^ CD3^+^ cells (T lymphocytes) (right panels). The data are representative of four independent experiments. For each sample a total number of 100,000 to 450,000 splenic cells of infected animals was collected and analyzed. The histograms show GFP expression in a total number of 1,700 dendritic cells, 1,500 macrophages, 1,600 granulocytes, 200,000 B lymphocytes, 3,000 NK cells, and 100,000 T lymphocytes. *Normalized frequency of cells is the number of cells falling within each bin of the histogram; there are 256 bins for each histogram.

To further dissect target cells of RVFV replication in *Ifnar1*-deficient mice, we used the recombinant virus rZHΔNSs-GFP that carries GFP in place of the NSs gene. We have shown previously that cells infected *in vitro* with rZHΔNSs-GFP are fluorescent upon excitation at 488 nm [24]. *Ifnar1*-deficient mice were either infected i.p. with 10^4^ PFU rZHΔNSs-GFP (N = 6) or mock-treated (N = 5). At 24 h post-infection, the spleen was dissected and single-cell suspensions were analyzed by flow cytometry for GFP expression. At this time point, 0.54% (range 0.14–1.53%) of the total hematopoietic cell population of the spleen from rZHΔNSs-GFP-infected mice expressed GFP whereas no GFP-positive cells were found in splenocytes after mock infection (Figure 7A). We examined the expression of GFP in various subsets of antigen presenting cells based on the surface expression patterns of CD45.2, CD11b, CD11c, Ly6G, CD19, CD3, NKp46, CD115 and MHCII class II by multicolor flow cytometric analysis. Among the CD11b^+^ CD115^+^ Ly6G^−^ (macrophages), CD11c^+^ CD11b^+^ MHC II^+^ (dendritic cells) [44] and CD11b^+^ CD11c^−^ Ly6G^+^ (granulocytes), on average 5.58% (range 2.15–8.71%), 4.5% (range 0.82–8.49%) and 1.96% (range 0.05–6.07%) cells expressed GFP, respectively (Figure 7C [left panels], B [right panels], and C [right panels], respectively). The percentage of GFP-expressing cells within the total cell population of spleen varied from one infected mice to another, indicating that the dynamics of RVFV infection progression was not identical in all individuals. However, each of the three subsets of immune cells was infected with the same efficiency in the different mice. This is shown by the fact that the ratio of GFP-expressing macrophages, dendritic cells or granulocytes was highly correlated with the ratio of GFP-expressing cells in the total cell population from the spleen (Pearson correlation coefficient 0.97, 0.85 and 0.79, respectively). These findings suggest a distinct pattern of susceptibility to infection by the RVFV-GFP for different immune cells in the following order: CD11b^+^ CD115^+^ Ly6G^−^ (macrophages)>CD11c^+^ CD11b^+^ MHC II^+^ (dendritic cells)>CD11b^+^ CD11c^−^ Ly6G^+^ (granulocytes). On average, less than 0.4% (range 0–1.19%) of NKp46^+^ CD3^−^ natural killer (NK) cells were positive for GFP (Figure 7E, left panel). Finally, GFP fluorescence was seen on average in only 0.25% (range 0.07–0.57%) of CD19^+^ CD3^−^ (B lymphocytes) cells and 0.20% (range 0.05–0.43%) NKp46^−^ CD3^+^ (T lymphocytes) cells (Figure 7D and E, right panels). Thus, at 24 h post-infection, RVFV replicated in cells of the myeloid lineage, primarily in mononuclear phagocytic cells, such as macrophages, dendritic cells and granulocytes.

**Figure 8:**
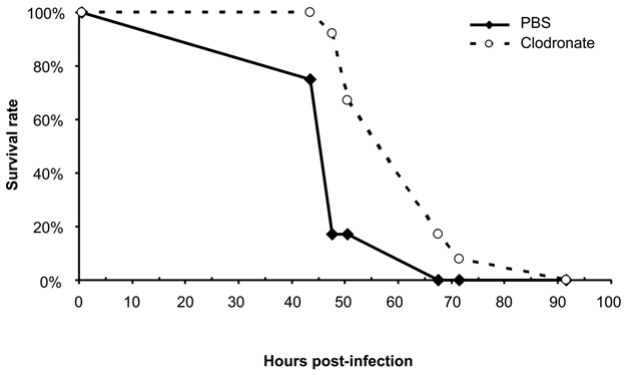
Survival curve of mice treated with clodronate-liposomes prior to infection with RVFV-GFP. Groups of twelve mice were injected i.p. and i.v. with either PBS-liposomes (PBSL) or clodronate-liposomes (CLL) prior to infection with 10^4^ PFU of the recombinant rZHΔNSs-GFP strain. The experiment was repeated once. Clodronate-liposome treated mice survived for a significantly longer period than mice treated with PBS-liposomes (*P*<0.0018).

To study the significance of virus replication in phagocytic cells *in vivo*, we injected intraperitoneally (i.p.) and intravenously (i.v.) clodronate-loaded liposomes (CLL) to *Ifnar1*-deficient mice prior to infection with RVFV. These liposomes are widely used to deliver clodronate to phagocytic cells, especially macrophages, and the accumulation of clodronate leads to irreversible metabolic damages, which will eventually result in apoptosis [45]. As reported previously [42], [43], the i.p. administration of CLL kills macrophages in the peritoneum and spleen of wild-type mice whereas i.v. administration affects mainly macrophages in the spleen and liver. We first analyzed the effect of CLL treatment on the phagocytic cell population of the blood and spleen of *Ifnar1*-deficient mice 24 h after i.p. and i.v. administration. Flow cytometric analysis was performed to compare the percentage of macrophages/monocytes, dendritic cells and granulocytes in samples from mice treated with CLL (N = 3) and PBS liposomes (PBSL) (N = 3). The CLL treatment resulted in a 23-fold reduction of CD11b^+^ CD115^+^ cells (monocytes) and a 6-fold reduction of CD11b^+^ F4/80^+^-expressing macrophages in the blood and spleen, respectively. In addition, CD11b^+^ CD11c^+^ F4/80^−^ cells (dendritic cells) were decreased 9-fold in the blood. By contrast, CD11b^+^ CD11c^−^ Ly6G^+^ cells (granulocytes) were not depleted in the blood and spleen, as previously reported [46]. Altogether this analysis showed that, 24 h after CLL treatment, blood monocytes and dendritic cells and spleen macrophages were efficiently depleted in *Ifnar1*-deficient mice whereas granulocytes were not affected. Next, CLL- or PBSL-treated mice were infected intradermally with 10^4^ PFU rZHΔNSs-hRLuc at 24 h after liposome administration. To investigate whether the clodronate treatment affected viral replication *in vivo*, we first observed PBSL- and CLL-administered infected mice using the imaging of whole bodies at 24 and 40 h post-infection. The profile of bioluminescence signals was similar in PBSL- and CLL-administered mice (N = 5 in each group) (data not shown). However, we observed weaker signals in CLL-administered mice. Indeed, the signals in the liver region were on average fifteen and four-fold lower at 24 and 40 h post-infection respectively in CLL- administered mice compared to PBSL-treated mice (*P*<0.05). We then measured the viraemia at 24 and 40 h post-infection in PBSL- and CLL-administered mice. At 24 h post-infection, the CLL-administered mice (N = 3) displayed lower blood plasma viral loads than the PBSL-treated mice (N = 3) (4.0×10^3^±2.9×10^3^ vs. 1.2×10^4^±2.7×10^3^ PFU/ml, Mann-Whitney test, *P* = 0.0495). Similar observations were made at 40 h post-infection (6.3×10^5^±3.7×10^5^ vs. 2.6×10^7^±2.2×10^7^ PFU/ml, Mann-Whitney test, *P* = 0.0495; *P* = 0.0039 when data from both time points were combined). Finally, the CLL-administered mice (N = 12) survived for a longer period of time after challenge than the PBSL-treated mice (N = 12) (Figure 8), suggesting that the depletion of monocytes/macrophages and dendritic cells prior to the viral infection affected the spreading and/or the containment of viral infection into cells of other organs. The mice showed no sign of encephalitis and succumbed to hepatitis. These observations confirmed the crucial role of monocytes/macrophages and dendritic cells during RVFV infection in *Ifnar1^−/−^* mice.

## Discussion

*In vivo* imaging studies using reporters, such as hRLuc and GFP, may provide a more complete picture of the spatiotemporal progression of a viral disease [47], [48]. In this study, we report the use of recombinant RVFV-hRLuc and RVFV-GFP strains to investigate the *in vivo* dynamics of RVFV infection progression in living mice and identify the virus-expressing cells. The recombinant viruses were generated by replacing the NSs gene with the reporter gene. Hence, these viruses were avirulent in immunocompetent mice when compared with wild-type virus but they were highly pathogenic in mice lacking interferon-α/β receptor, enabling to use them for pathogenesis studies in this mouse model. We were able to detect luciferase reporter expression at early stages of infection in the main known sites of viral replication, the liver, the spleen, the thymus and the brain [18], [21], [49], [50], [51]. The pancreas appeared as an unexpected site of virus replication. Using *ex vivo* imaging and histological examination, we primarily identified macrophages infiltrating the adipose tissue surrounding the pancreas as primarily virus-expressing cells. Similarly, RVFV-expressing macrophages were identified in the stroma of the ovary. The RVFV-GFP confirmed the importance of macrophages as specific host cells for the virus in *Ifnar1^−/−^* mice. It further allowed the identification of dendritic cells and granulocytes as target cells for RVFV replication. Interestingly, only a low number of B-, T- and NK-cells expressed the GFP reporter. Viral antigens have been previously detected in mononuclear phagocytic cells and dendritic cells in the lymph nodes, spleen and thymus from infected wild-type mice [21] and in macrophages in the lymph nodes from infected rats [51]. Interestingly, Smith and colleagues [21] also noticed that lymphocytes did not appear stained with the RVFV antibody in agreement with our observations. Although RVFV replication in the human macrophage-like cell line U937 [52] and in cultured peritoneal macrophages from susceptible rats [53] have been previously documented, this is, to our knowledge, the first study to evaluate the infection rates of various subsets of cells of the myeloid lineage *in vivo*. Because the level of fluorescent GFP directly reflects the extent of transcription and replication of the recombinant virus, we assume that the virus is highly expressed in GFP-positive cells. However, since macrophages, dendritic cells and granulocytes are able to uptake cell debris, it is possible that some of these cells are GFP-positive following phagocytosis of debris of RVFV-GFP-infected cells *in vivo*.

Phagocytic cells function as pathogen sensors. Macrophages and neutrophils provide the first line of defense following infections. Macrophages and dendritic cells are antigen presenting cells and play a crucial role in the establishment of the adaptive immune response. Infection with RVFV-GFP showed that phagocytic cells are also target cells for RVFV. We investigated the importance of the *in vivo* interaction between phagocytic cells and RVFV. We treated *Ifnar1*-deficient 129S2/SvPas mice with CLL to deplete the population of phagocytic cells, and showed that, following intradermal infection with RVFV, the depleted mice allowed reduced RVFV replication compared to control mice, as assessed both by *in vivo* imaging and viral titration from blood samples. Accordingly, CLL-treated mice displayed enhanced survival time compared with control mice, indicating that phagocytic cells are involved in the pathogenesis of RVF. Altogether, our data suggest that during the initial stages of infection of *Ifnar1^−/−^* mice, the virus replicates inside macrophages and dendritic cells. On the other hand, since the RVFV replicates in diverse cell types in peripheral tissues, the infection may progress rapidly and lead to acute hepatitis and death.

RVFV is thought to be transmitted primarily by bites of infected mosquitoes, by direct contact with infected body fluids or through airborne transmission. It has been confirmed that exposure of mice to aerosols containing RVFV is able to induce infection [54]. Following inoculation into a dermal site, RVFV-hRLuc expression was seen in the draining lymph node which became the main site of replication early after infection while the virus was still weakly detected into the abdominal cavity. Later, virus spread and caused severe hepatitis within 69 h post-infection. Following intranasal inoculation, virus replicated in the lung where it caused pneumonia within 48 h post-infection. Its dissemination to the abdominal cavity was rapid and mice succumbed at 69 h post-infection. Thus, typical routes of exposure were associated with clear differences in the spatial and temporal progression of RVFV and caused delayed death compared with i.p. inoculation.

Type I interferons (IFNs) are essential elements during host antiviral defense [55]. Both recombinant RVFV strains inoculated i.p. were able to kill *Ifnar1*-deficient 129S2/SvPas mice within 2 days whereas wild-type 129S2/SvPas mice survived infection, indicating that a functional IFN-α/β pathway is critical for the protection of mice from fatal infection with these attenuated viruses. We showed that the recombinant viruses could replicate in known target tissues and cells of RVFV. It is not clear whether in the absence of the IFN-α/β receptor, the reporter RVFV can replicate in tissues and cells that are not normally susceptible to infection with a fully virulent RVFV in wild-type mice. Hence, we infected wild-type 129S2/SvPas with the virulent RVFV ZH548 strain and observed infected macrophages in the spleen and pancreaticoduodenal lymph node. However, we failed to identify the peritonitis seen repeatedly in recombinant RVFV-infected *Ifnar1^−/−^* mice. This suggests that, in *Ifnar1^−/−^* mice, cells of the macrophage lineage displayed an increase susceptibility to RVFV compared to wild-type mice. The high susceptibility of cells of the macrophage/dendritic lineage to viral infection in the absence of a functional type I IFN system has been previously observed. An increased infection of cells of the macrophage/dendritic lineage was observed in *Ifnar1^−/−^* mice infected with either the Sindbis virus [56], or the mouse hepatitis virus [57]. Similarly, macrophages showed the greatest increase in susceptibility among the different splenocyte populations in West Nile virus-infected *Ifnar1^−/−^* mice compared to wild-type mice [58]. More generally, an increase in the replication of viruses in tissues and cells normally susceptible to virus infection has been previously observed in *Ifnar1^−/−^* mice. Coxsackievirus replicated dramatically in the liver of *Ifnar1*-deficient compared with wild-type mice [59]. Finally, previous investigation of poliovirus replication sites in infected *Ifnar1^−/−^* mice expressing the human poliovirus receptor showed that nontarget tissues became potentially permissive for virus infection when IFNα/β signaling was disrupted [60]. Therefore, the fact that *Ifnar1^−/−^* mice inoculated with NSs-deficient RVFV strains develop acute hepatitis and eventually die, as wild-type mice infected with a virulent RVFV strain, does not mean that the exact mechanisms of the cellular pathogenesis are the same in *Ifnar1^−/−^* and wild-type mice. Thus, although *Ifnar1^−/−^*mice have proven to be a tractable system in which to study the progression of RVFV infection *in vivo*, the immunocompromised nature of this mutant strain remains a limitation in translating these results directly to wild-type mice. Additional work needs to be done to develop similar whole-organ imaging and flow cytometry analysis in immune-competent mice. Further studies involving the use of fully virulent RVFV – i.e. carrying the NSs gene – which express a reporter gene, might allow us to give a comprehensive picture of the dynamics of natural infection in mammals. Our work provides the basis for the use of bioluminescent and fluorescent RVFV to study the effects of specific mutations in the viral genome and of host genetic factors on the tissue tropism and replication kinetics in living mice.
